# Short-Term Effect in Soil Microbial Community of Two Strategies of Recovering Degraded Area in Brazilian Savanna: A Pilot Case Study

**DOI:** 10.3389/fmicb.2021.661410

**Published:** 2021-06-09

**Authors:** Priscila Jane Romano Gonçalves Selari, Luiz Ricardo Olchanheski, Almir José Ferreira, Tiago do Prado Paim, Guido Calgaro Junior, Flavio Lopes Claudio, Estenio Moreira Alves, Darliane de Castro Santos, Welington Luiz Araújo, Fabiano Guimarães Silva

**Affiliations:** ^1^Laboratory of Microbiology, Department of Biology, Instituto Federal de Educação, Ciência e Tecnologia Goiano (Federal Institute of Education, Science and Technology Goiano), Ceres, Brazil; ^2^Laboratory of Microbiology, Department of Structural and Molecular Biology and Genetics, State University of Ponta Grossa (UEPG), Ponta Grossa, Brazil; ^3^Laboratory of Molecular Biology and Microbial Ecology, Department of Microbiology, Institute of Biomedical Sciences, University of São Paulo (USP), São Paulo, Brazil; ^4^Laboratory of Education in Agriculture Production, Instituto Federal de Educação, Ciência e Tecnologia Goiano (Federal Institute of Education, Science and Technology Goiano), Iporá, Brazil; ^5^Laboratory of Agricultural Chemistry, Instituto Federal de Educação, Ciência e Tecnologia Goiano (Federal Institute of Education, Science and Technology Goiano), Rio Verde, Brazil; ^6^Laboratory of Plant Tissue and Culture, Instituto Federal de Educação, Ciência e Tecnologia Goiano (Federal Institute of Education, Science and Technology Goiano), Rio Verde, Brazil

**Keywords:** crop-livestock integrated system, land use, metagenomics, soil microbiome, sustainability

## Abstract

The Brazilian Cerrado is a highland tropical savanna considered a biodiversity hotspot with many endemic species of plants and animals. Over the years, most of the native areas of this biome became arable areas, and with inadequate management, some are nowadays at varying levels of degradation stage. Crop-livestock integrated systems (CLIS) are one option for the recovery of areas in degradation, improving the physicochemical and biological characteristics of the soil while increasing income and mitigating risks due to product diversification. Little is known about the effect of CLIS on the soil microbial community. Therefore, we perform this pilot case study to support further research on recovering degraded areas. The bacterial and fungal soil communities in the area with CLIS were compared to an area under moderate recovery (low-input recovering - LI) and native savanna (NS) area. Bacterial and fungal communities were investigated by 16S and ITS rRNA gene sequencing (deep rRNA sequencing). Ktedonobacteraceae and AD3 families were found predominantly in LI, confirming the relationship of the members of the Chloroflexi phylum in challenging environmental conditions, which can be evidenced in LI. The CLIS soil presented 63 exclusive bacterial families that were not found in LI or NS and presented a higher bacterial richness, which can be related to good land management. The NS area shared 21 and 6 families with CLIS and LI, respectively, suggesting that the intervention method used in the analyzed period brings microbial diversity closer to the conditions of the native area, demonstrating a trend of approximation between NS and CLIS even in the short term. The most abundant fungal phylum in NS treatment was Basidiomycota and Mucoromycota, whereas Ascomycota predominated in CLIS and LI. The fungal community needs more time to recover and to approximate from the native area than the bacterial community. However, according to the analysis of bacteria, the CLIS area behaved differently from the LI area, showing that this treatment induces a faster response to the increase in species richness, tending to more accelerated recovery. Results obtained herein encourage CLIS as a sustainable alternative for recovery and production in degraded areas.

## Introduction

The Cerrado (Brazilian savanna) is the second largest biome in Brazil, covering approximately 24% of the country’s territory. The climate of the region is marked by high temperatures and long periods of drought. The soils are naturally acidic, poor in nutrients, and rich in aluminum, with tropical savanna vegetation and distinct physiognomies ranging from pastures to forests (Souza et al., 2016). The most abundant type of vegetation is the Cerrado *stricto sensu* which is characterized by a forest savanna with low and twisted trees, shrubs, and large grasslands (Araujo et al., 2012). The “Cerradão” is a forest floristically similar to the Cerrado *stricto sensu*, with trees ranging from 8 to 15 m tall and from 50 to 90% cover (Dall’Agnol et al., 2016).

Despite being considered a global hotspot of biodiversity, approximately 50% of the Cerrado native areas have been transformed into mechanized agriculture, forestry, and intensive livestock areas over the years (López-Poma et al., 2020). The inadequate management of production systems can lead to the degradation of these areas and reduce their productivity. There are several strategies for recovering these areas under degradation, and the choice between these strategies relies on the stage of degradation, financial resources available, and other soil and topographic limitations for mechanization.

The crop-livestock integrated system (CLIS) involves intercropping, crop rotation, and succession with livestock grazing in the same area. This system aims to improve agricultural productivity and recover degraded land, due to improvements in soil physical, chemical, and biological characteristics (Lisboa et al., 2014; Sarto et al., 2020). CLIS enables a higher yield and more stable income due to the diversity of products generated (mainly, cereals, legumes and animal products, meat, and milk), which can reduce the pressure for deforestation in other areas (Damian et al., 2020). However, the first year of CLIS implantation involves a series of soil disturbances like limestone and gypsum application and plowing with different methodologies. This management is required to prepare the soil physically and chemically to crop seeding. On the other hand, these soil managements can negatively impact soil biological conditions (Sándor et al., 2020).

Other recovering strategies propose minor soil interventions with lower inputs (fertilizer, machinery, and money), taking more time to reach the full recovery of the area. These strategies could preserve soil biological content, which would help in the recovering process.

Microorganisms are essential for soil productivity and fertility because they perform several important functions, such as decomposition of organic matter, nutrient solubilization, and cycling, and synthesis of phytohormones and antimicrobial compounds, thus promoting plant growth and protection (Nannipieri et al., 2017). Different land uses and management systems can modify the structure and functioning of the soil microbial community (Hermans et al., 2020), thus affecting plant productivity.

There are several studies reporting changes in the microbial diversity in Brazilian Cerrado soil due to land use transformations (Castro et al., 2016; Souza et al., 2016; Araujo et al., 2017; Castañeda and Barbosa, 2017; Vieira et al., 2018; Silva et al., 2019). Studies of the soil of the Brazilian Cerrado under CLIS using crop-dependent methods revealed a reduction in the microbial diversity and enzymatic activity in the forest component of the CLIS compared to the crop rows. In addition, the microbial biomass and abundance of bacteria and fungi were higher in the native Cerrado than in the CLIS and conventional monoculture system (Sarto et al., 2020). In contrast, an CLIS in temperate agroforestry systems increased the abundance of various bacteria and fungi in the soil compared to the conventional monoculture system, increasing soil fertility (Beule et al., 2020). The soil microbiome in Cerrado has been studied under different points of view, including the impact of agriculture on taxonomic and functional microbial diversity (Souza et al., 2016), mining areas under revegetation (Vieira et al., 2018), high yield and degraded pasture areas (Silva et al., 2019), different vegetation physiognomies (Castro et al., 2016; Araujo et al., 2017), and even in the search for new lipolytic enzymes with biotechnological potential (Istvan et al., 2018). However, little is known about the short-term effect of CLIS implementation on the soil microbial community.

Thus, the objective of this pilot case study was to evaluate the short-term effects of recovering degraded areas in the composition and structure of soil bacterial and fungal communities. The present study evaluated this soil biological condition in fields under recovery through CLIS and under a moderate intervention recovering comparing to native vegetation in the Brazilian savanna (Cerrado).

## Materials and Methods

### Characterization of the Study Area

**FIGURE 1:**
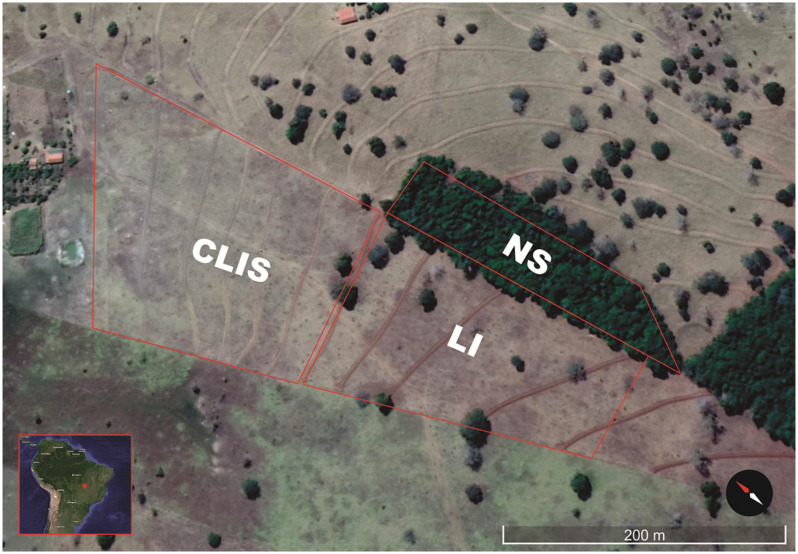
Image of UEPE Boa Esperança, Iporá, Goiás, Brazil, in June of 2018. The demarcated fields represent the treatments: NS - native savanna, LI - low-input recovering, and CLIS - crop-livestock integrated system.

The study was conducted at the “Boa Esperança” Teaching, Research, and Extension Unit (UEPE Boa Esperança for its acronym in Portuguese; 16°26′34.951″N 51°1′2.539″E) of the Institutional Project for Integrated Agricultural Production Systems of the Goiano Federal Institute (IF GOIANO) located in Iporá city, state of Goiás, Brazil (Figure 1). The soil was classified as typical dystrophic Dark Red Latosol, moderate A horizon, mixed texture (IUSS Working Group WRB, 2015), and flat relief (<6% slope), at 495 m of altitude.

The climate of the region is classified as tropical savanna (AW, Köppen), being 24.7°C the annual average temperature and 1,369 mm the rainfall average. The dry season is from May to September (winter) and the rainfall average is 62 mm. The rainy season is from October to April (summer) and the rainfall average is 1,307 mm^1^.

### Design and Characterization of Treatments

Three areas with different land use strategies were evaluated: (a) recovery of an area in degradation via CLIS, (b) recovery of an area in degradation using a moderate intervention strategy (low-input recovering, LI), and (c) native Cerrado control area (native savanna, NS).

The CLIS field received 1.8 Mg.ha^–1^ of dolomitic limestone (85% of Relative Power of Total Neutralization), and 4.00 Mg.ha^–1^ of poultry litter applied on the surface. After, chisel plow at 0.25 m depth and 0.6 m spacing was applied. The remaining plants in the area (predominantly *Urochloa brizantha* and *Urochloa humidicola*) were desiccated with 1,440 g of glyphosate.ha^–1^. Seven days after desiccation, a triple intercropping with corn (*Zea mays* cv. “AG1051”), pigeon pea (*Cajanus cajan* cv. “Super N”), and Tamani grass (*Megathyrsus maximus* cv. “BRS Tamani”) were simultaneously sowed. The sowing was made with a 5-row mechanical seed drill using three maize rows intercalated with two pigeon pea rows. An additional fine seeds distribution system in the same machine sowed Tamani grass (5 kg of pure viable seeds.ha^–1^). The pigeon pea had a final population of 28,250 plants.ha^–1^ and maize had 67,000 plants.ha^–1^. The fertilization used was 200 kg.ha^–1^ of 8-41-5 (nitrogen-phosphorus-potassium). Topdressing fertilizations with N were performed in the corn vegetative stages of two (V2) and four (V4) fully expanded true leaves using 46 kg.ha^–1^ of N (100 kg.ha^–1^ of urea) in each operation.

The intercropped forage was harvested for silage production in February 2018 (90 days after sowing). The forage collected was composed of all biomass available (corn, pigeon pea, and Tamani grass) in 2 m^2^. The material was ground and homogenized, then ensiled in micro-silos (polyvinyl chloride material, 10 cm × 30 cm) for anaerobic fermentation. After 60 days, the micro-silos were opened, and the material was dried at 65°C by 72 h. The dry matter (DM) content was determined, and the DM yield per hectare was calculated. The dry material was grounded in a 1-mm Wiley mill to determine the levels of calcium (Ca), phosphorus (P), crude protein (CP), crude fiber (CF), ether extract (EE), mineral matter (MM), total digestible nutrients (TDN), acid detergent fiber (ADF), and neutral detergent fiber (NDF) (AOAC, 1995).

Thirty days after harvesting, the area was divided into 12 paddocks (3,125 m^2^). A total of 15 animals (Girolando dairy cows) were used in a rotational grazing scheme according to target sward height management, 35 cm pre-grazing, and 25 cm post-grazing (Tesk et al., 2020).

The LI field also received 1.8 Mg.ha^–1^ of dolomitic limestone superficially spread followed by chisel plow at 0.25 m depth and 0.6 m spacing. The forage species (*Urochloa brizantha*) present in the field was maintained and used for livestock grazing during the following year. The area was divided into eight paddocks (4,687 m^2^ each) and grazed by 15 animals (Girolando dairy cows) in a rotational grazing scheme following the same management (Tesk et al., 2020).

The Native savanna area sampled is located at the side of the two recovering fields. The collection points were 100 to 160 m away, which ensure the same soil source and type at all three sites evaluated here.

### Soil Sampling and Analyses

The soil samples for chemical analyses were composed of five subsamples collected in each area (NS, CLIS, and LI) at 0–20 cm depth that were homogenized prior to measurement. These samples were used for the characterization of the soil before the beginning of the recovering process (collected in June 2017) and after the first year of recovery management (July 2018). The average soil textures for clay, silt, and sand were 24, 12, and 64% (0–20 cm), respectively. The average chemical composition was 4.6 pH (CaCl_2_), 0.75 Ca (cmolc dm^–3^), 0.3 Mg (cmolc dm^–3^), 1.05 Ca + Mg (cmolc dm^–3^), 0.1 Al (cmolc dm^–3^), 2.55 H + Al (cmolc dm^–3^), 3.8 CEC (cmolc dm^–3^), 1 P Melich (mg dm^–3^), 70 K (mg dm^–3^), 14 Organic Matter (g.kg^–1^), 11.5 Aluminium saturation (%), and 31% of Base saturation (V%).

A new soil collection was performed in July 2018 for DNA extraction of the microbial community, collecting four samples in each site (NS, CLIS, and LI). Each sample consisted of three subsamples obtained within a radius of 3 m, which were duly homogenized. The collection points of the samples between treatments were less than 160 m apart. The samples for microbial diversity analyses were at 5–10 cm depth.

### Soil DNA Extraction and Next-Generation Sequencing

The sampled soil was sieved (2-mm-mesh sieve) and placed in 50-mL tubes, which were properly sealed, identified, and immediately placed in liquid nitrogen. In the laboratory, the samples were stored in a freezer at −80°C until processing.

Total DNA was extracted from each of the 12 soil samples using the DNeasy PowerSoil Kit (Qiagen, San Francisco, CA, United States) following the manufacturer’s instructions. For bacteria, the V3-V4 regions of the bacterial 16S rRNA gene were amplified using the universal primers 515F (Parada et al., 2016) and 806R (Apprill et al., 2015) with an Illumina adaptor. For fungi, the primers ITS1F and ITS2 (Hoggard et al., 2018) with an Illumina adaptor were used to amplify the Internal Transcribed Sequence 1 (ITS-1) of the ribosomal gene cluster. The PCR products were purified using the QIAquick PCR Purification Kit (Qiagen, San Francisco, CA, United States) before performing agarose gel electrophoresis. The concentrations of the purified amplicon products were determined by NanoDrop^TM^ 1000 spectrophotometry (Thermo Fisher Scientific, Waltham, MA, United States) and sequenced on an Illumina MiSeq PE3000 platform (Illumina, San Diego, CA, United States) and CD Genomics (Shirley, New York, NY, United States).

### Soil Microbial Community Analyses

The sequence data demultiplexed were imported into the Quantitative Insights into Microbial Ecology 2 (QIIME2, version 2021.2.0; Caporaso et al., 2010). The Divisive Amplicon Denoising Algorithm 2 (DADA2; Callahan et al., 2016) was used to quality filter, trim, denoise, merge the pairs of reads, and remove chimeric sequences (15 nucleotides were removed from the forward and reverse reads, according to visual inspection of the quality at each nucleotide). The taxonomic assignment was carried out using the feature-classifier plugin against SILVA SSU 138 non-redundant database for bacterial taxonomy (Quast et al., 2013; Bokulich et al., 2018), and UNITE 8.2 (Abarenkov et al., 2020) for fungi taxonomy.

Species richness, diversity (Shannon and Simpson indices), and Pielou evenness indices were estimated for each of the samples from the ASV table using the software R v.4.0.2 (R Core Development Team, 2019) through the “vegan” package (Oksanen et al., 2013). MANOVA was conducted to explore the differences between land management. Means were compared with the “ScottKnott” package (Jelihovschi et al., 2014). Normality and homogeneity of the residual distribution were inspected. Non-metric multidimensional scaling (NMDS) of the weighted Unifrac distance matrix was used to visualize the bacterial community structure. The NMDS analyses were performed using the packages “ggord” (Beck, 2016), “vegan” and “factoextra” (Kassambara and Mundt, 2017) and the graphical representation used the “ggplot2” package (Wickham, 2009). The Permutational Multivariate Analysis of Variance (PERMANOVA) was performed using the package “vegan.” Venn diagrams were made using the R VennDiagram package to analyze each taxonomic level shared among the NS, CLIS, and LI areas (Chen and Boutros, 2011).

The crude reads were deposited at the National Center for Biotechnology Information (NCBI) with BioProject accession number PRJNA669331.

## Results

The results of the soil chemical analysis in the first year of recovering process are shown in Table 1. These results represent a characterization of the chemical composition of the soil in each area and are shown to demonstrate the contrasting soil conditions in each land management system.

**TABLE 1:** Analysis of soil chemical attributes in July 2018 (one year after recovering process started) at a depth of 0–20 cm in the different treatments evaluated (NS - native savanna, LI - low-input recovering, CLIS - crop-livestock integrated system).

**Attributes**	**NS**	**CLIS**	**LI**
pH (CaCl_2_) (un)	4.4	5.0	5.4
Ca (cmolc dm^–3^)	0.4	1.5	1.4
Mg (cmolc dm^–3^)	0.2	0.4	0.4
Ca + Mg (cmolc dm^–3^)	0.6	1.9	1.8
Al (cmolc dm^–3^)	0.3	0.0	0.0
H + Al (cmolc dm^–3^)	2.8	2.1	1.5
CEC (cmolc dm^–3^)	3.6	4.2	3.4
P (Melich I) (mg dm^–3^)	3.0	4.0	2.0
K (cmolc dm^–3^)	0.20	0.22	0.11
K (mg dm^–3^)	80.0	88.0	44.0
Organic Matter (g kg^–1^)	23.0	12.0	12.0
Sat. Al (M%)	27.0	0.0	0.0
Base Saturation (V%)	23.0	51.0	56.0
Ca/Mg	2.0	3.8	3.5
Ca/CEC	11.1	35.7	41.2
Mg/CEC	5.6	9.5	11.8
K/CEC	5.7	5.4	3.3

Crop-livestock integrated system represented the crop-livestock integrated system using a triple intercropping of corn, pigeon pea, and grass. The forage yield was 7.2 Mg.ha^–1^ of corn, 1.7 Mg.ha^–1^ of pigeon pea, and 4.9 Mg.ha^–1^ grass, totaling 13.8 Mg.ha^–1^ of total forage, on a dry matter basis. Therefore, the silage dry matter was composed of 52.2% of corn, 12.3% of pigeon pea, and 35.5% of Tamani grass. The chemical composition of the silage was 0.3, 0.1, 7.8, 28.2, 3.5, 5.8, 64.1, 42.6, and 59.9% for Ca, P, CP, CF, EE, MM, TDN, ADF, and NDF, respectively. These results demonstrate the capacity of CLIS to produce high-quality forage in a great amount.

### Bacterial Soil Community

About 2.3 million reads were obtained from the sequencing of the 16S rRNA. After applying quality filters, merging reads, and chimera removal, the 873,043 bacterial sequences were grouped into 12,299 ASVs distributed in 944 taxonomic groups at level 7 (species). From species-level classification, 733 species were found in the CLIS sample, representing the most diverse sample, followed by the LI sample (563 species), and NS (494 species). Among the species observed, 33.5% (316) corresponded to the bacterial core present in all sampled areas, regardless of the treatment. In another hand, 229, 82, and 103 OTUs were exclusive for CLIS, LI, and NS areas, respectively. Unclassified sequences, which corresponded to 0.012% of the total, were excluded from the analyses.

In order to verify the difference in the abundance of taxonomic groups among the different land managements, a non-metric multidimensional scaling analysis (NMDS) was applied. Phylum, Class, Order, and Family were able to group the samples according to land management, verified by PERMANOVA (*p* = 0.001).

The sequencing of the 16S rRNA allowed the identification of 35 phyla (Supplementary Figure 1). The first component of NMDS analyses separated NS from the other two. The second component demonstrated some variability inside CLIS and LI. The NS area shared four phyla exclusively with the CLIS and no phylum was shared between NS and LI and between LI and CLIS, demonstrating a closer relation of CLIS with NS. Among the phyla, two were represented by the Archaea domain, with a predominance of Proteobacteria (22.8%), Actinobacteria (22.7%), Chloroflexi (14.5%), and Acidobacteriota (13.5%). Native savanna areas were associated with higher Acidobacteriota (17.2%), Verrucomicrobiota (13.7%), and Planctomycetota (8.5%), compared to the other cultivated areas. Higher Actinobacteriota were found in CLIS and LI. The highest Chloroflexi abundance was observed in LI and the lowest in NS, while CLIS had an intermediate position. Chloroflexi was the only phyla that differentiate between the three land management systems (Supplementary Figure 1).

Analyzing class-level taxonomy, there were found 76 classes, the most abundant being Alphaproteobacteria (19.3%), Actinobacteria (10.6%), Acidobacteriaceae (8.7%), Verrucomicrobiae (8.1%), Thermoleophila (7.9%), and Ktedonobacteria (6.6%). The classes Verrucomicrobiae (13.4%), Acidobacteria (12.5%), Planctomycetes (8.1%), and Gammaproteobacteria (5%), were significantly more abundant in the NS area than in the CLIS (5.8, 7.1, 4.8, and 2.7%, respectively) and LI treatments (5.1, 6.5, 5.4, and 2.7%, respectively) (*p* = 0.05). These classes were also positively correlated with the first component in NMDS. Thermoleophilia, Actinobacteria, and Bacilli were more predominant in CLIS and LI and negatively correlated with the first component in NMDS. Ktedonobacteria were found significantly more abundant in LI (*p* = 0.05) and positively correlated with the second dimension in NMDS (Supplementary Figure 2).

In NS, the orders Chthoniobacterales (12.2%) and Acidobacteriales (6%) were significantly more abundant than in the CLIS (5.1 and 3.9%, respectively) and LI (4.6 and 3.4%, respectively) treatments. Solirubrobacterales were less abundant in the NS area. NMDS based on bacterial orders showed a higher separation of CLIS and LI in the second dimension, with only one observation of LI at the positive side. Ktedonobacterales abundance was higher in LI samples and negatively correlated with NMDS2, explaining the separation of LI to CLIS (Supplementary Figure 3). Rhizobiales had the highest positive correlation with NNMDS2, which can indicate higher abundance in CLIS than LI. CLIS had the highest number of exclusive orders and shared 16 and 15 orders exclusively with NS and LI, respectively, demonstrating the higher diversity in the soil of this land management system.

**FIGURE 2:**
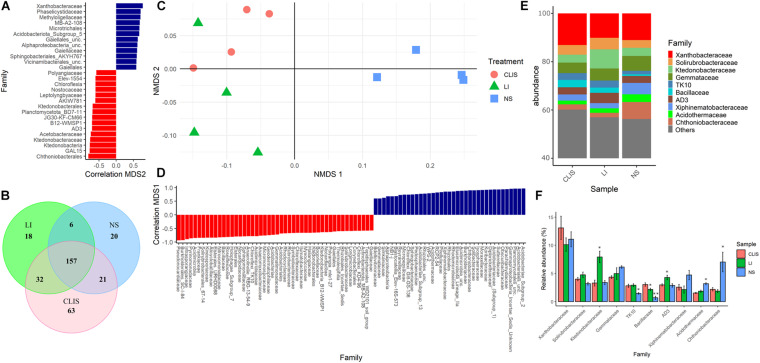
Evaluation of the bacterial community (Family) in the native savanna (NS), low-input recovering (LI), and crop-livestock integrated system (CLIS) areas obtained by sequencing of 16S rRNA genes. **(A)** Family with significant correlation with the second component of NMDS. **(B)** Venn diagram obtained from Family data. **(C)** Individual coordinates from NMDS results. **(D)** Family with significant correlation with the first component of NMDS. **(E)** Relative abundance of bacterial families in each area. **(F)** Percentage of relative abundance of bacterial families in each area. Asterisks represent significance according to the Scott-Knott method *p* < 0.05.

Non-metric multidimensional scaling analysis of soil bacterial community showed that dissimilarities at the family level were driven by sampling area (Figure 2), very similar to the results based on the order. Ktedonobacteraceae and AD3 had higher abundance in LI compared to NS and CLIS, being negatively correlated with NMDS2 (Figures 2A,F).

The NS treatment shared 21 families with CLIS and six families with LI. The CLIS and LI areas shared 32 families. Twenty families were exclusive to the NS, 18 families were exclusive to LI, and 63 families were exclusive to the CLIS (Figure 2B). Xanthobacteraceae was the most abundant family (Figure 2E) found in all the studied areas (NS = 11%; CLIS = 13%, and LI = 10.1%) and contributes positively with the second dimension in NMDS, representing the separation between CLIS and LI (Figures 2A,C). Relative abundances of Chthoniobacteraceae and Acidothermaceae were higher in NS (7 and 3.2%, respectively) than CLIS (2.2 and 1.5%) and LI (1.8 and 1.8%). On the other hand, TK10 and Bacillaceae were less abundant in NS than CLIS and LI areas.

### Fungal Soil Community

About 2.6 million reads were obtained from the sequencing of the ITS region. After applying quality filters, merging reads, and chimera removal, the 1,716,917 fungal sequences were grouped into 12,610 ASVs distributed in 1094 taxonomic groups at level 7 (species). From species-level classification, 671 species were found in the NS, representing the most diverse sample, followed by the LI sample (505 species), and CLIS (499 species). Among the species observed, 15.3% (167) corresponded to the bacterial core present in all sampled areas, regardless of the treatment. In another hand, 149, 151, and 380 species were exclusive for CLIS, LI, and NS areas, respectively. Of the reads approved in the quality index, 26.4% were not classifiable from the database used and were not included in other analyses.

Abundances of Basidiomycota and Mucoromycota were higher, while Ascomycota was less abundant in NS compared to CLIS and LI areas (*p* = 0.05), both phyla were positively correlated with the first dimension, related to separation between NS and the others (Supplementary Figure 4). Ascomycota and Glomeromycota were negatively correlated with the second dimension of NMDS (Supplementary Figure 4), representing the separation between CLIS and LI.

According to the taxonomic analysis of classes, Agaricomycetes was the most abundant class in NS (64%). CLIS and LI showed less than 20% of the abundance of the Agaricomycetes. Eurotiomycetes, Sordariomycetes, and Dothideomycetes were less abundant in NS (Supplementary Figure 5).

**FIGURE 3:**
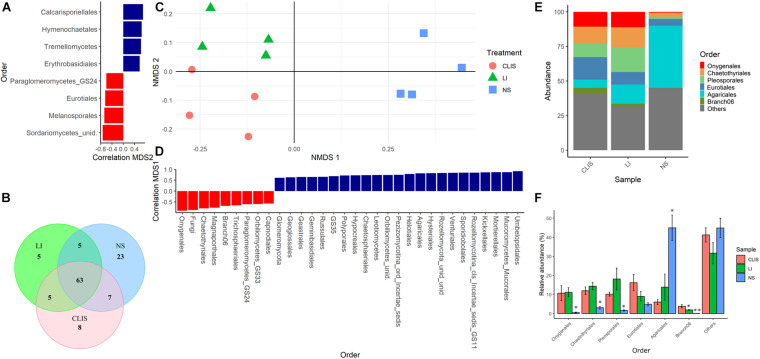
Evaluation of the fungal community (Order) in the native savanna (NS), low-input recovering (LI), and crop-livestock integrated system (CLIS) areas obtained by sequencing of ITS rRNA genes. **(A)** Order with significant correlation with the second component of NMDS. **(B)** Venn diagram obtained from Order data. **(C)** Individual coordinates from NMDS results. **(D)** Order with significant correlation with the first component of NMDS. **(E)** Relative abundance of fungal orders in each area. **(F)** Percentage of relative abundance of fungal families in each area. Asterisks represent significance according to the Scott-Knott method *p* < 0.05.

Non-metric multidimensional scaling analysis for fungal orders showed a clear separation among areas (Figures 3A,C,D). The NS area shared seven orders with CLIS and five orders with LI. The CLIS and LI areas shared five orders among themselves. Twenty-three orders were exclusive to the NS, five to LI, and eight to CLIS (Figure 3B). Agaricales was predominant in NS (45%). Onygenales, Chaetothyriales, Pleosporales, and Branch 06 were less abundant in NS (0.5, 3, 1.6, and 0.1%, respectively) when compared to CLIS (10.7, 12, 18, and 3.8%, respectively) and LI (11.1, 14.3, 18.1, and 1.9%, respectively) (Figures 3E,F). Eight fungal orders were significantly correlated with the second dimension, which divided the CLIS and LI. Sordariomycetes, Melanosporales, Eurotiales, and glomeromycetes were higher in CLIS while Calcarisporiellales, Hymenochaetales, Tremellomycetes, and Erythrobasidiales were higher in LI (Figure 3A).

The NMDS with fungal family data separated the three areas, the first dimension separated NS from others and the second dimension separated LI from CLIS (Supplementary Figure 6). In NS, sequences of unidentified families were more abundant (30.7%), followed by Hygrophoraceae (31.7%), which represented only 0.9% in CLIS and 7.75% in LI. Herpotrichiellaceae was less abundant in NS (1.78%) compared to the other areas (CLIS = 7.8%; LI = 8.6%). Those families were important to the separation between CLIS and NS in the second component of NMDS (Supplementary Figure 6).

The abundances of the microbial communities obtained in each sample are shown in Supplementary Figures 7–10. In general, the abundances were consistent across repetitions.

### Diversity Index of Soil Microbial Community

The bacterial diversity (Shannon-Wiener and Simpson indices) and Pielou evenness indices showed no differences among the sampled areas. However, the bacterial richness was higher in the CLIS than in the LI and NS. In the NS area, the fungal diversity and Pielou evenness indices were smaller than in CLIS and LI, while the fungal richness was higher in native savanna in comparison to CLIS and LI treatments (Table 2).

**TABLE 2:** Analysis of richness (Chao1, ACE), diversity (Shannon and Simpson), and evenness (Pielou) in the 16S rRNA genes of bacteria and the ITS region of fungi, as well as the number of OTUs, observed in each treatment (NS, native savanna, LI, low-input recovering, and CLIS, crop-livestock integrated system).

**Sequence**	**Treatment**	**Shannon**	**Simpson**	**Pielou**	**Richness**
16S	NS	4.87 ± 0.26^a^	0.98 ± 0.005^a^	0.85 ± 0.01^a^	305.50 ± 71.73^b^
	CLIS	5.17 ± 0.15^*a*^	0.98 ± 0.003^a^	0.85 ± 0.02^a^	449.00 ± 20.61^a^
	LI	5.09 ± 0.14^a^	0.98 ± 0.002^a^	0.87 ± 0.01^a^	345.00 ± 54.67^b^
ITS	NS	3.15 ± 0.45^b^	0.86 ± 0.07^b^	0.54 ± 0.08^b^	346.25 ± 52.08^a^
	CLIS	3.76 ± 0.21^a^	0.94 ± 0.01^a^	0.68 ± 0.02^a^	256.50 ± 38.51^b^
	LI	3.81 ± 0.33^a^	0.94 ± 0.02^a^	0.69 ± 0.04^a^	253.00 ± 42.60^b^

*Means followed by the standard deviation with the same letter are not significantly different according to the Scott-Knott method *p* < 0.05.*

## Discussion

Since the experiment was conducted on a commercial farm, there were limitations to develop an experimental design with traditional repetitions. Therefore, we provided here a pilot case study to explore the potential impact of CLIS management on soil biodiversity and microbial structure, and the results obtained here were representative for the studied place, which is inserted in the Cerrado biome.

The productivity achieved in three months, which was verified through phytotechnology and bromatological analyses of the forage, was included in this work to prove that the forage produced at CLIS is of higher quality compared to traditional production systems. Thus, it reinforces our hypothesis that CLIS promotes soil regeneration, besides being able to represent economic gains to the producer (Salton et al., 2014). The results obtained were similar to those observed by Ligoski et al. (2020) that studied the same intercropping system. These authors showed that this intercropped silage can be a good ruminant feedstuff and decrease animal methane emissions due to ruminal fermentation.

The culture-independent analysis revealed that the predominant phyla of bacteria were Proteobacteria, Actinobacteria, and Acidobacteria, which varied in abundance between the sampled areas. The differences in relative abundances suggest the influence of the different conditions of each area, such as soil pH, nutrients, and vegetation. Moisture, temperature, soil composition, and pH shape the bacterial community in the soil, and pH is one of the main influencers of bacterial diversity and richness (Alsharif et al., 2020). Zhalnina et al. (2014) also state that pH directs the structure of the soil microbial community because it affects the availability of nutrients for microorganisms.

Proteobacteria, Actinobacteria, and Acidobacteria are often abundant phyla in Cerrado soil (Silva et al., 2019). Among the Proteobacteria classes, there is a prevalence of the class Alphaproteobacteria in the Cerrado (Araujo et al., 2017). Actinobacteria are often found in arid and semiarid environments. The members of this phylum are versatile and can grow under extreme conditions of salinity, temperature, radiation, pH, and low water availability (Alsharif et al., 2020). The species of the phylum Actinobacteria are often related to hostile environmental conditions and weathering (O’Brien et al., 2019), which are conditions that can be found in CLIS and LI and explain the high incidence of this phylum in those areas.

The phylum Acidobacteria was found in greater abundance in the NS, which can be explained by the more acidic pH found in this area since the representatives of this group have tolerance to lower pH (Araujo et al., 2017), which had a pH 4.4 (Table 1). Verrucomicrobia was another phylum with a higher incidence in the native area, which includes oligotrophic bacteria found in soil regions that are poor in nutrients, conditions found in native soils of Cerrado (Silva et al., 2019). Firmicutes had a higher incidence in CLIS and LI than NS (Supplementary Figure 1). This phylum contains representatives resistant to unfavorable conditions (Araujo et al., 2017). In addition, the class Bacilli, which has important species involved in the promotion of plant growth through different mechanisms (Chaudhry et al., 2017), was more abundant in CLIS and LI, demonstrating ecological changes and possible improvements in soil functions.

The phylum Chloroflexi was less abundant in NS and CLIS than LI. Representants of this phylum are markedly found in anaerobic habitats where play a role in fermentation and degradation of organic compounds to support their growth and that of other bacterial populations (Speirs et al., 2019). It was expected to find more anaerobic conditions in the LI area since is not yet fully recovered and presents greater degradation than the other areas. In terms of the abundance of this phylum, in particular, it seems that CLIS with the intervention is getting closer to the NS area. This idea was reinforced by the Venn diagram, which shows four phylum sharing between CLIS and NS.

The family Chthoniobacteraceae (phylum Verrucomicrobia) was most abundant in NS, while CLIS and LI showed abundances three times lower of this family. Granada et al. (2019) related the high incidence of members of the family Chthoniobacteraceae in areas with higher levels of organic matter, as seems to be the case when comparing NS with CLIS and LI. A similar pattern was observed for the family Acidothermaceae, which was most frequent in NS than CLIS and LI. In turn, the abundances of Ktedonobacteraceae and AD3 were greater in LI. Ktedonobacteraceae belongs to the phylum Chloroflexi and are often found in a higher abundance in extreme environments (Yabe et al., 2017). AD3 is related to sandy soils, highly weathered soils, and soils poor in organic C (Brewer et al., 2019). The members of this family are commonly adapted to conditions of nutritional limitation and present characteristics such as spore formation, synthesis and storage of carbohydrates, and use of carbon monoxide as an energy source (Brewer et al., 2019). Considering that the LI area is not yet fully recovered and presents greater degradation than the other areas, this would explain the higher incidence of AD3. Associated with this we can highlight that NS shared a larger number of families with CLIS than with LI area. And thus, although the CLIS treatment did not present the same parameters as the native area, there is evidence that the intervention method used in the analyzed period brings microbial diversity closer to the conditions of the native area.

The variations in the abundances of the families found in the studied areas reinforce the idea of a seed microbial bank, which refers to the ability of an organism to decrease its metabolic activity in the face of adverse conditions while still living in that environment and later being reactivated under favorable conditions (Lennon and Jones, 2011). This would explain the higher incidence of certain families in the areas, since they showed differences in the management, in addition to explaining the persistence of rare groups. Our results provide evidence that the different taxa respond differently to the types of management, which affects the functioning and restoration of the ecosystem.

The CLIS had the highest bacterial richness among the studied areas. This result can be explained by the fact that the area contains cultivated plants in addition to animals. Different plants and root exudates attract different groups of microorganisms to the rhizosphere (Alsharif et al., 2020; Pascale et al., 2020).

According to the NMDS analyses, the three areas analyzed were grouped in different clusters. There was a more pronounced separation of the NS from the two recovered areas (CLIS and LI) when analyzing bacterial data. The fungal data showed a more pronounced separation of LI from the other two.

The difference between the CLIS and LI areas shows that the compositions of the fungal and bacterial communities differed. Regarding the analysis of fungal sequences, the NS showed a dominance of the phylum Basidiomycota. The phylum Ascomycota predominated in CLIS and LI, which suggests that degradation in the areas could lead to the loss of basidiomycetes, and this niche is occupied by ascomycete fungi. The prevalence of ascomycetes has been correlated with human activity and the high tolerance to environmental stresses by representatives of the phylum (Souza et al., 2016). The change in agricultural management of Cerrado soil caused changes in the structure of the fungal community of the soil, mainly due to changes in soil properties, such as nutritional levels, CEC, and base saturation (Valadares-Pereira et al., 2017).

Studies on microbial diversity in Cerrado *stricto sensu* areas have shown that the predominant fungal phyla in dry seasons are Ascomycota and Basidiomycota (Castro et al., 2016), which is in agreement with the results obtained in this study. However, the relative abundance of basidiomycetes in the rainy season increases, while the relative abundance of ascomycetes decreases, compared to that in the dry season; this demonstrates the strong effect of soil water content on the fungal community structure (Castro et al., 2016). The conditions in the NS might have contributed to its higher moisture levels than those in the LI and CLIS treatments, which contributed, together with other factors, to a greater development of basidiomycetes in NS.

The dominant family in NS was Hygrophoraceae, whose representatives are primarily found in undisturbed grassland habitats and are much rarer or absent in grasslands subject to agricultural intensification, which can characterize them as ecological indicators (Halbwachs et al., 2018). Thus, it is evident that the fungal community needs more time to recover and to approximate from the native area than the bacterial community, mainly because those are more sensitive to environmental changes (Genevieve et al., 2019). This explains also the higher fungal richness and the smaller fungal diversity in the NS area compared to CLIS and LI. Although there is a greater richness in NS, the evenness is lower (confirmed by the diversity and Pielou indexes). That is, in a native environment, certain species are prevalent, which is not being observed in the regenerating areas. It is likely due to the kinetics of nutrients and altered environmental conditions for the recovery of the areas, which makes the “disturbance” in the areas (or lack of environmental stability) prevent the development of certain species at the expense of others.

## Conclusion

This study evaluated the taxonomic composition of the bacterial and fungal communities of the soil in CLIS compared to LI and NS areas. The land-use type affected the taxonomic diversity of the soil microbiota. The analyses of the microbial community showed that after one year of intervention in the degraded area, the CLIS area already showed a more accelerated recovery than the LI area, mainly marked by the bacterial communities. The results presented here contribute to a better understanding of the factors that help to model the microbial communities in strategic systems used for the recovery of degraded areas and encourage the use of CLIS for those areas.

## Data Availability Statement

The datasets generated for this study can be found in NCBI under BioProject accession number PRJNA669331.

## Author Contributions

PS, TP, GJ, FC, EA, and DS contributed to the conception and design of the study. GJ, FC, and EA conducted the field work and the soil sampling. PS performed the DNA extraction and soil and DNA analysis. LO and AF conducted the bioinformatics. LO ran the statistical analysis. PS, LO, and EA wrote the manuscript. TP, EA, DS, WA, and FS contributed to the manuscript revision. FS contributed to the funding acquisition and supervision. All authors read and approved the submitted manuscript.

## Conflict of Interest

The authors declare that the research was conducted in the absence of any commercial or financial relationships that could be construed as a potential conflict of interest.

## Footnotes

1 https://pt.climate-data.org/america-do-sul/brasil/goias/ipora-43430/
