# Supplementary material for: Short-Term Effect in Soil Microbial Community of Two Strategies of Recovering Degraded Area in Brazilian Savanna: A Pilot Case Study
Source: Front Microbiol. 2021 Jun 9;12:661410. doi: 10.3389/fmicb.2021.661410 (PMC8221397; doi:10.3389/fmicb.2021.661410)
Supplement: Supplementary file 1 [file Data_Sheet_1.PDF]

Supplementary Material

Short-term effect in soil microbial community of two strategies of recovering degraded area in Brazilian savanna

Priscila Jane Romano Gonçalves Selari*, Luiz Ricardo Olchanheski, Almir José Ferreira, Tiago do Prado Paim, Guido Calgaro Junior, Flavio Lopes Claudio, Estenio Moreira Alves, Darliane de Castro Santos, Welington Luiz Araújo, Fabiano Guimarães Silva.

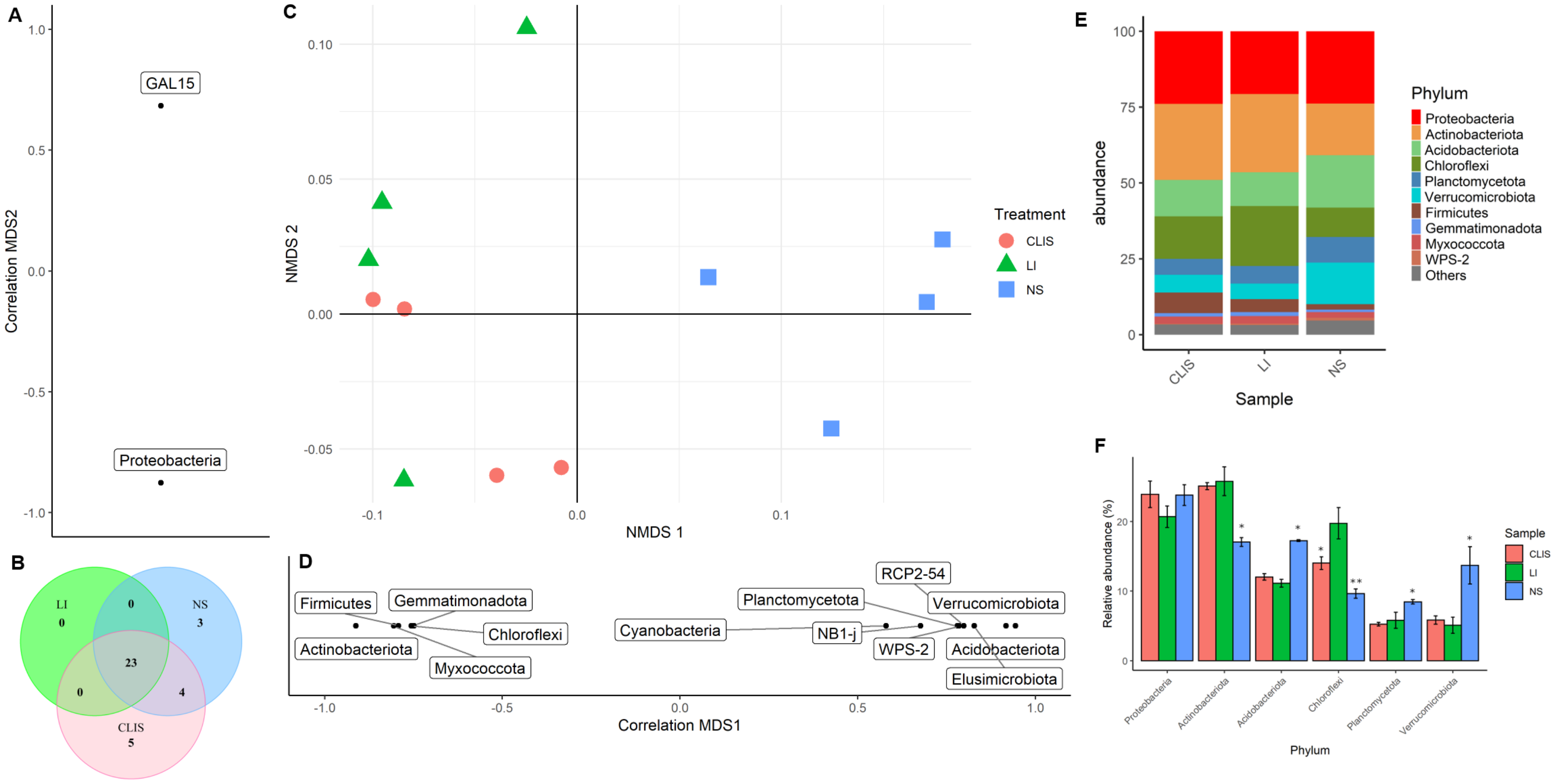

Supplementary Figure 1. Evaluation of the bacterial community (Phylum) in the native savanna (NS), low-input recovering (LI), and crop–livestock integrated system (CLIS) areas obtained by sequencing of 16S rRNA genes. (**A**) Phyla with significant correlation with the second component of NMDS (**B**) Venn diagram obtained from Phylum data. (**C**) Individual coordinates from NMDS results **(D)** Phyla with significant correlation with the first component of NMDS **(E)** Relative abundance of bacterial phyla in each area **(F)** Percentage of relative abundance of bacterial phyla in each area. Asterisks represent significance according to the Scott-Knott method $p < 0.05$.

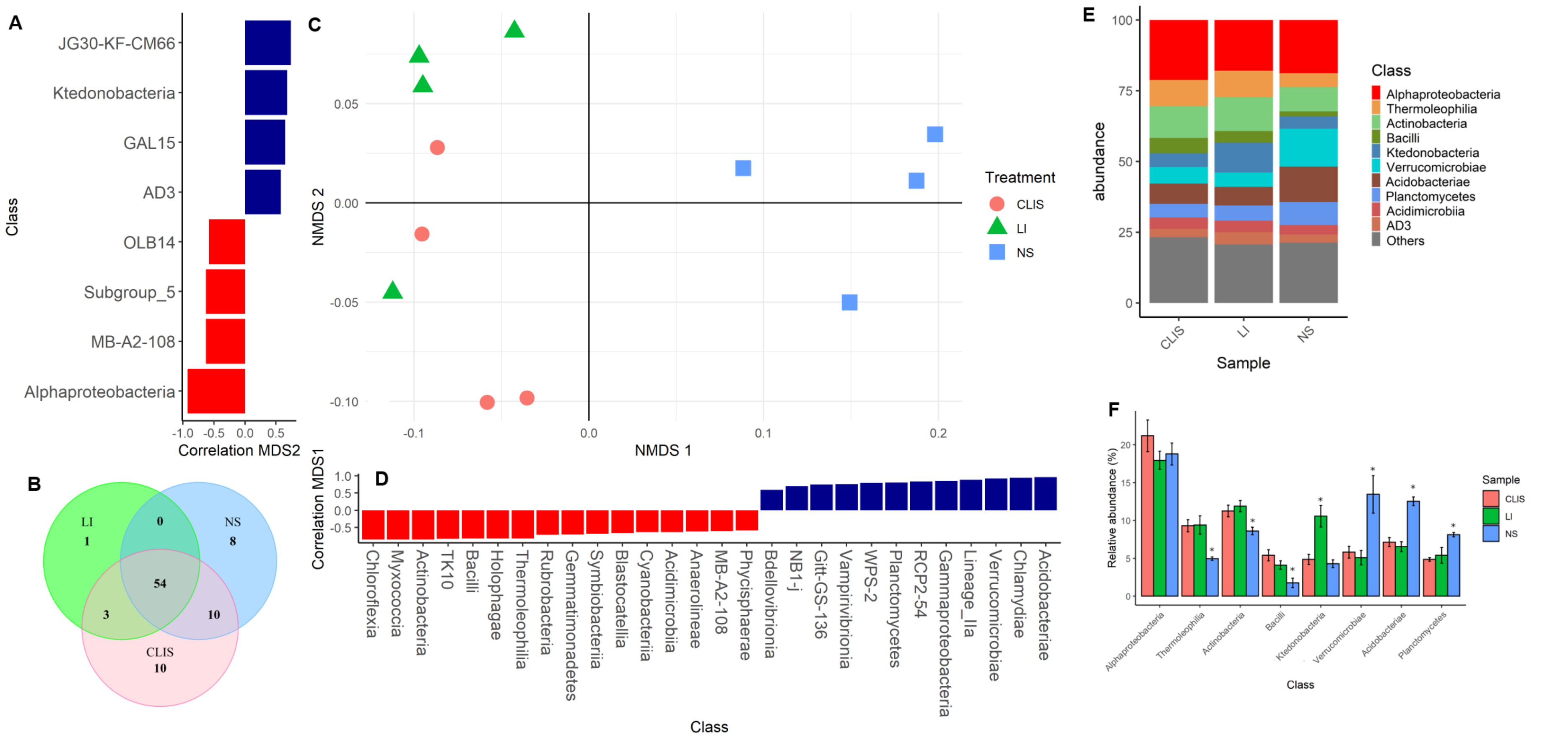

Supplementary Figure 2. Evaluation of the bacterial community (Class) in the native savanna (NS), low-input recovering (LI), and crop–livestock integrated system (CLIS) areas obtained by sequencing of 16S rRNA genes. (**A**) Classes with significant correlation with the second component of NMDS (**B**) Venn diagram obtained from Class data. (**C**) Individual coordinates from NMDS results **(D)** Classes with significant correlation with the first component of NMDS **(E)** Relative abundance of bacterial classes in each area **(F)** Percentage of relative abundance of bacterial classes in each area. Asterisks represent significance according to the Scott-Knott method $p < 0.05$.

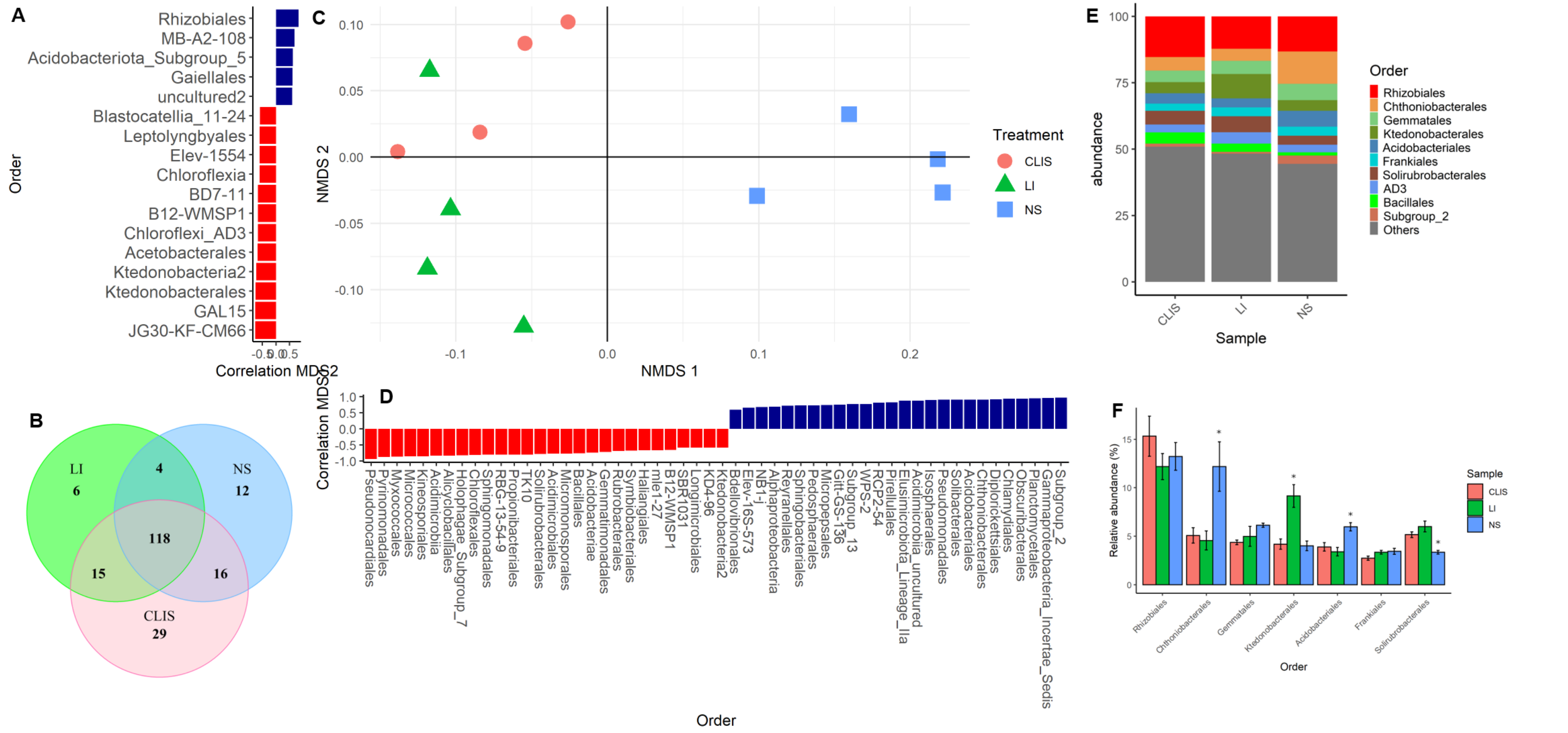

Supplementary Figure 3. Evaluation of the bacterial community (Order) in the native savanna (NS), low-input recovering (LI), and crop–livestock integrated system (CLIS) areas obtained by sequencing of 16S rRNA genes. (**A**) Orders with significant correlation with the second component of NMDS (**B**) Venn diagram obtained from Order data. (**C**) Individual coordinates from NMDS results **(D)** Orders with significant correlation with the first component of NMDS **(E)** Relative abundance of bacterial orders in each area **(F)** Percentage of relative abundance of bacterial orders in each area. Asterisks represent significance according to the Scott-Knott method $p < 0.05$.

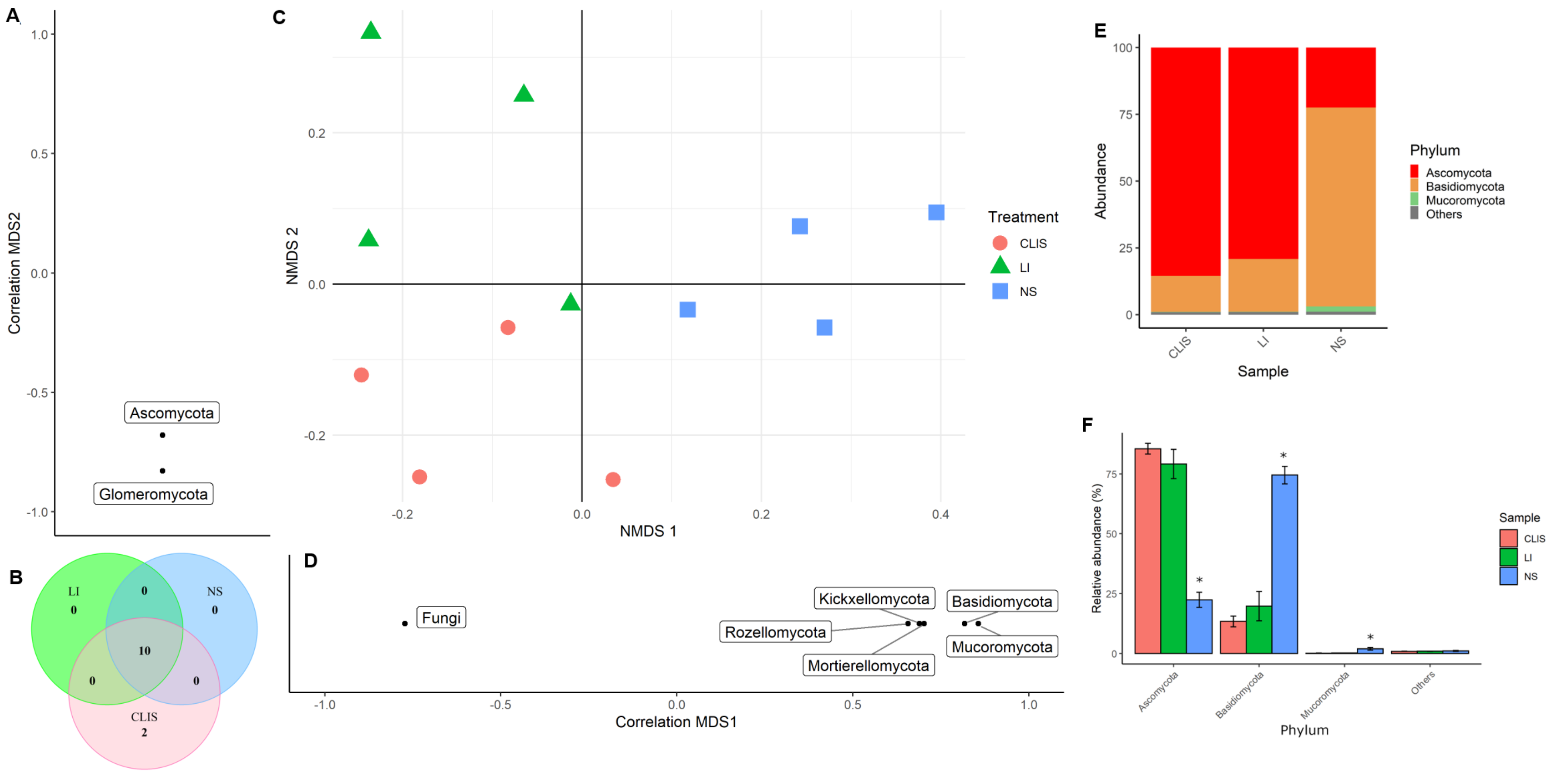

Supplementary Figure 4. Evaluation of the fungal community (Phylum) in the native savanna (NS), low-input recovering (LI), and crop–livestock integrated system (CLIS) areas obtained by sequencing of ITS rRNA genes. (**A**) Phyla with significant correlation with the second component of NMDS (**B**) Venn diagram obtained from Phylum data. **(C)** Individual coordinates from NMDS results **(D)** Phyla with significant correlation with the first component of NMDS **(E)** Relative abundance of fungal phyla in each area **(F)** Percentage of relative abundance of fungal phyla in each area. Asterisks represent significance according to the Scott-Knott method $p < 0.05$.

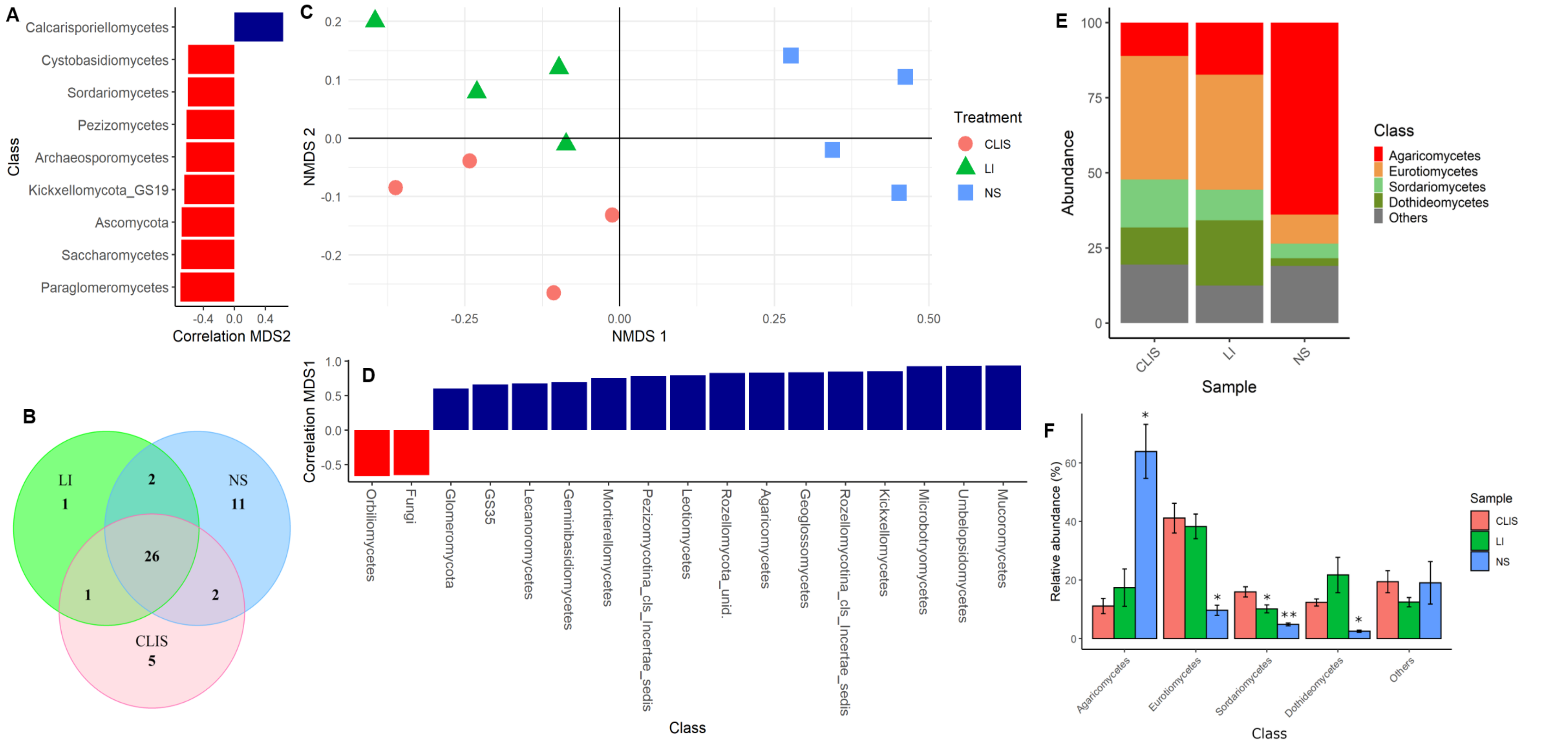

Supplementary Figure 5. Evaluation of the fungal community (Class) in the native savanna (NS), low-input recovering (LI), and crop–livestock integrated system (CLIS) areas obtained by sequencing of ITS rRNA genes. (**A**) Classes with significant correlation with the second component of NMDS (**B**) Venn diagram obtained from Class data. (**C**) Individual coordinates from NMDS results **(D)** Classes with significant correlation with the first component of NMDS **(E)** Relative abundance of fungal classes in each area **(F)** Percentage of relative abundance of fungal classes in each area. Asterisks represent significance according to the Scott-Knott method $p < 0.05$.

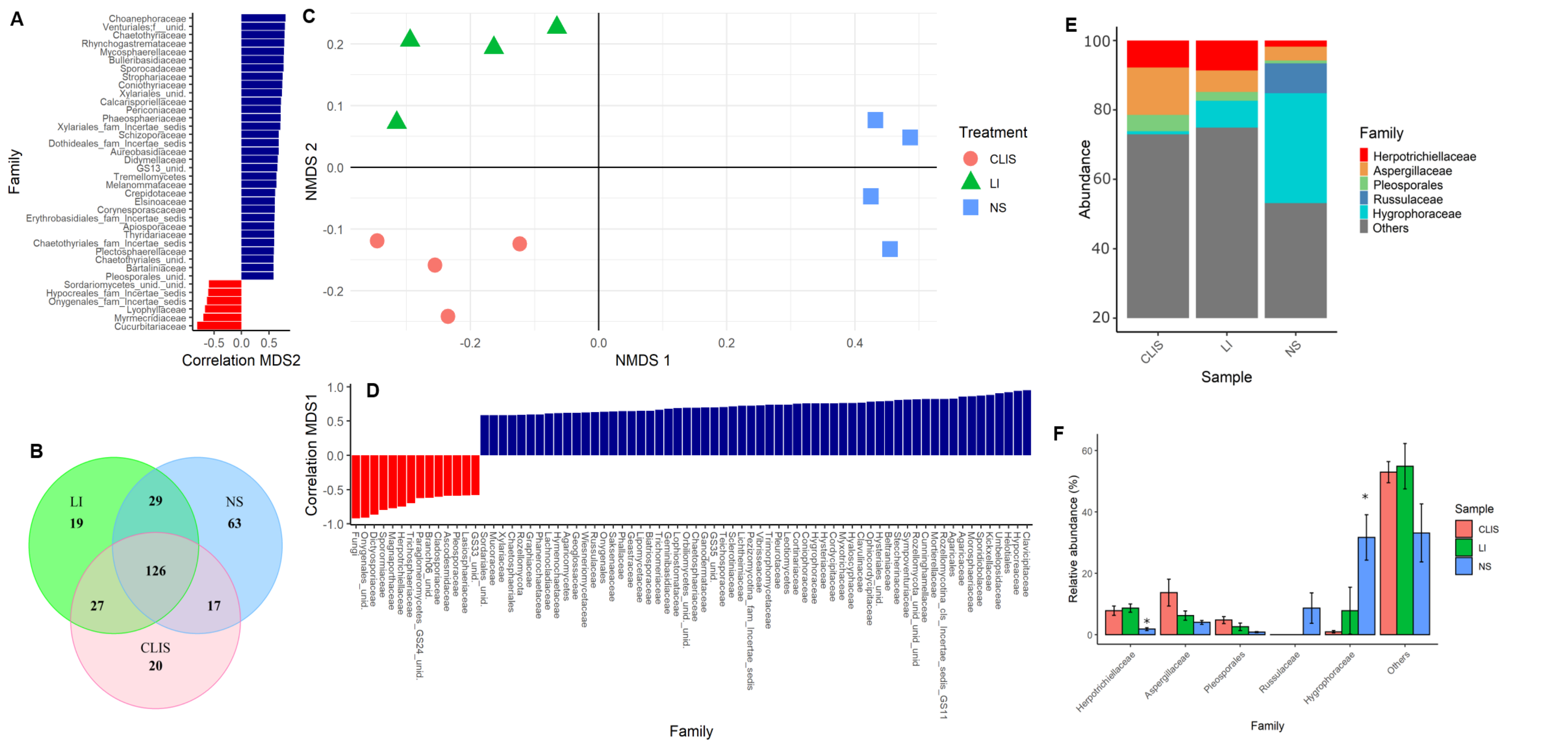

Supplementary Figure 6. Evaluation of the fungal community (Family) in the native savanna (NS), low-input recovering (LI), and crop–livestock integrated system (CLIS) areas obtained by sequencing of ITS rRNA genes. (**A**) Families with significant correlation with the second component of NMDS (**B**) Venn diagram obtained from Family data. **(C)** Individual coordinates from NMDS results **(D)** Families with significant correlation with the first component of NMDS **(E)** Relative abundance of fungal families in each area **(F)** Percentage of relative abundance of fungal families in each area. Asterisks represent significance according to the Scott-Knott method $p < 0.05$.

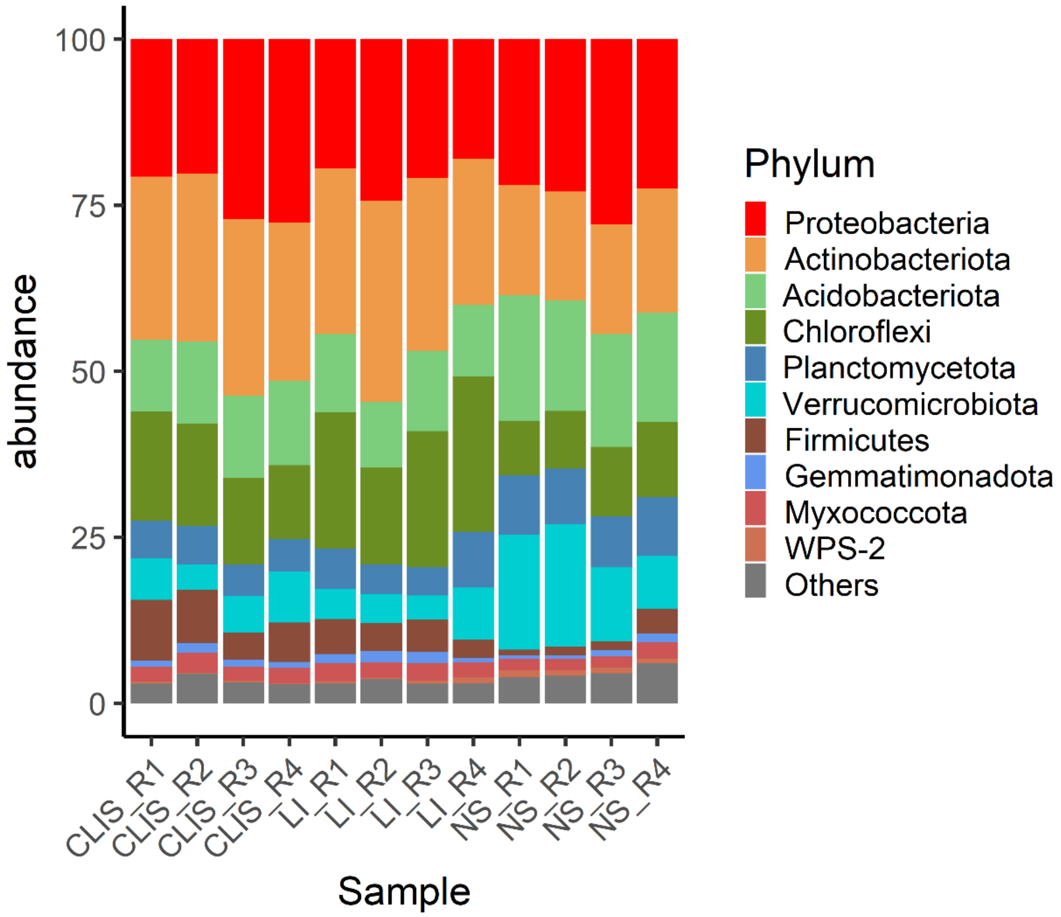

Supplementary Figure 7. Relative abundance of bacterial phyla in each treatment: Native Savanna (NS), Low-Input recovering (LI) e Crop-Livestock Integrated System (CLIS) obtained by sequencing of 16S rRNA genes. R1 to R4 represent replicates.

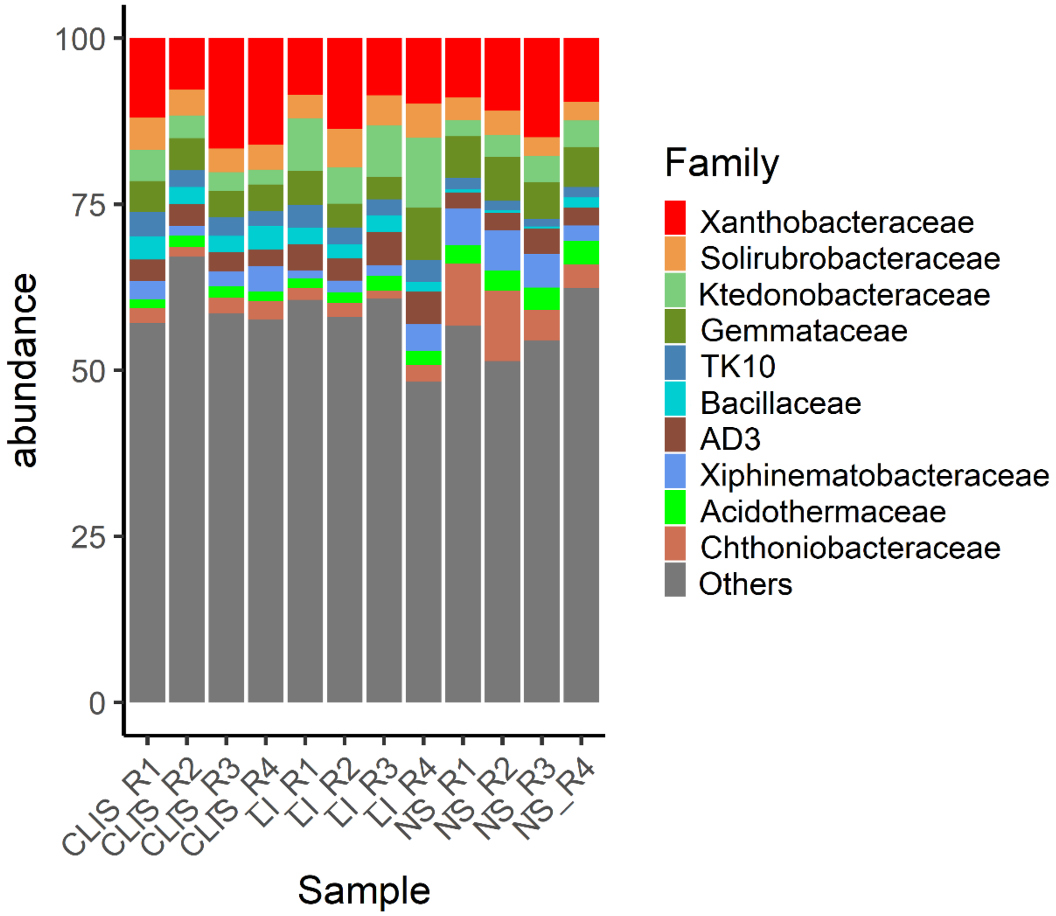

Supplementary Figure 8. Relative abundance of bacterial family in each treatment: Native Savanna (NS), Low-Input recovering (LI) e Crop-Livestock Integrated System (CLIS) obtained by sequencing of 16S rRNA genes. R1 to R4 represent replicates.

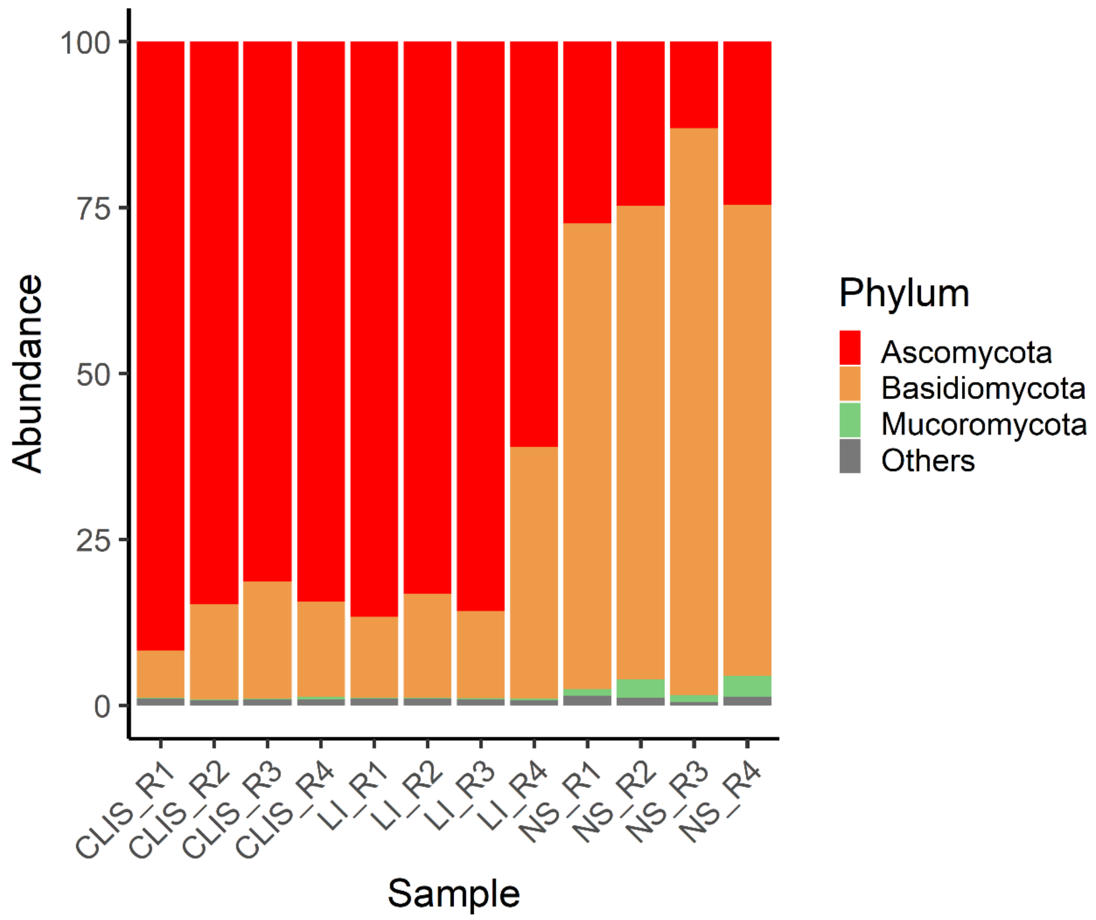

Supplementary Figure 9. Relative abundance of fungal phyla in each treatment: Native Savanna (NS), Low-Input recovering (LI) e Crop-Livestock Integrated System (CLIS) obtained by sequencing of ITS rRNA genes. R1 to R4 represent replicates.

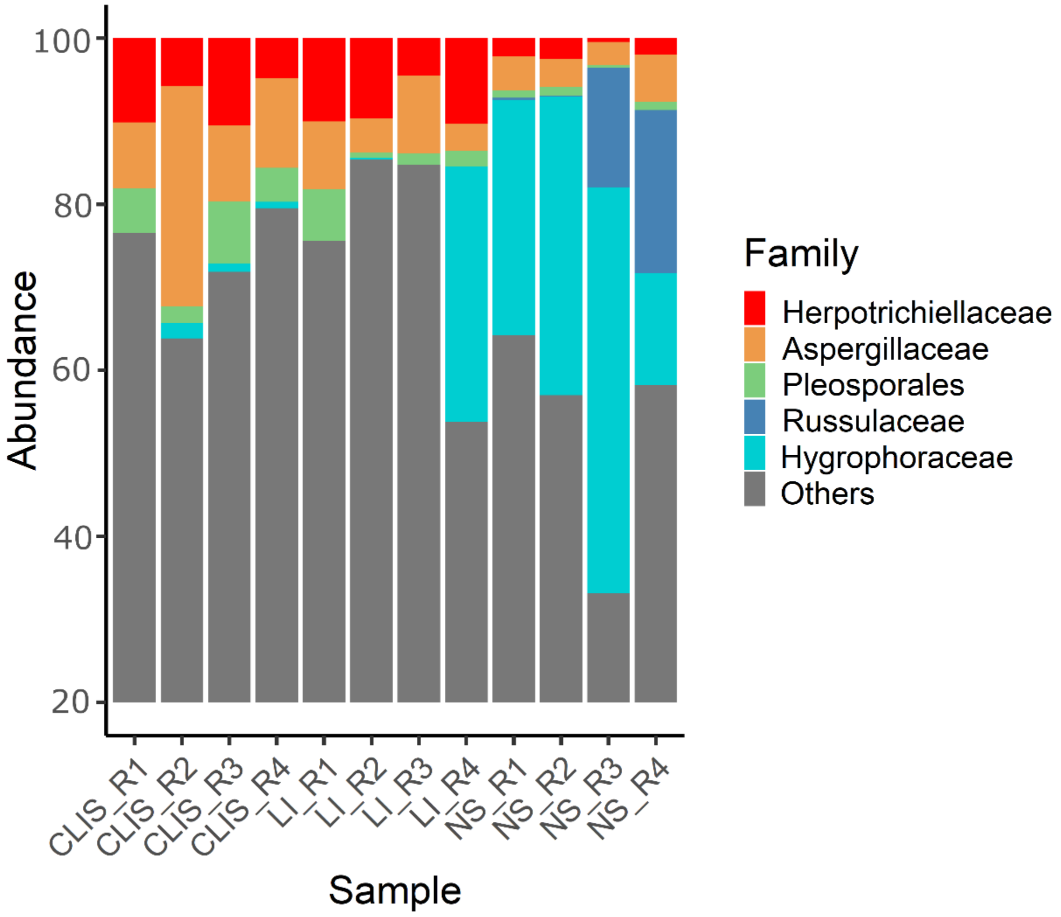

Supplementary Figure 10. Relative abundance of fungal families in each treatment: Native Savanna (NS), Low-Input recovering (LI) e Crop-Livestock Integrated System (CLIS) obtained by sequencing of ITS rRNA genes. R1 to R4 represent replicates.
